# Anomalous asthma and chronic obstructive pulmonary disease Google Trends patterns during the COVID-19 pandemic

**DOI:** 10.1186/s13601-020-00352-9

**Published:** 2020-11-02

**Authors:** Bernardo Sousa-Pinto, Enrico Heffler, Aram Antó, Wienczyslawa Czarlewski, Anna Bedbrook, Bilun Gemicioglu, G. Walter Canonica, Josep M. Antó, João Almeida Fonseca, Jean Bousquet

**Affiliations:** 1 MEDCIDS–Department of Community Medicine, Information and Health Decision Sciences, Faculty of Medicine, University of Porto, Porto, Portugal; 2 CINTESIS–Center for Health Technology and Services Research, University of Porto, Rua Dr. Plácido da Costa, 4200-450 Porto, Portugal; 3 Personalized Medicine, Asthma & Allergy, Humanitas Clinical and Research Center, IRCCS, Rozzano, Italy; 4 Department of Biomedical Sciences, Humanitas University, Pieve Emanuele, Milan, Italy; 5 MASK-Air, Montpellier, France; 6 Medical Consulting Czarlewski, Levallois, France; 7 MACVIA-France, Montpellier, France; 8 Department of Pulmonary Diseases, Cerrahpasa Faculty of Medicine, Istanbul University-Cerrahpasa, Istanbul, Turkey; 9 ISGlobAL, Centre for Research in Environmental Epidemiology (CREAL), Barcelona, Spain; 10 Universitat Pompeu Fabra (UPF), Barcelona, Spain; 11 CIBER Epidemiología y Salud Pública (CIBERESP), Barcelona, Spain; 12 Charité, Universitätsmedizin Berlin, Humboldt-Universität Zu Berlin, Berlin, Germany; 13 Department of Dermatology and Allergy, Comprehensive Allergy Center, Berlin Institute of Health, Berlin, Germany; 14 Centre Hospitalier Universitaire, Montpellier, France

**Keywords:** Asthma, Chronic diseases, Chronic obstructive pulmonary disease, COVID-19, Google trends

## Abstract

**Background:**

An increase in online searches on health topics may either mirror epidemiological changes or reflect media coverage. In the context of COVID-19, this is particularly relevant, as COVID-19 symptoms may be mistaken for those of respiratory disease exacerbations. Therefore, we aimed to assess Internet search patterns on asthma and chronic obstructive pulmonary disease (COPD) in the context of COVID-19, as compared to searches on other chronic diseases.

**Methods:**

We retrieved Google Trends (GTs) data on two respiratory (asthma and COPD) and three non-respiratory (diabetes, hypertension, and Crohn’s disease) chronic diseases over the past 5 years (up to May 31, 2020). For 54 countries, and for each disease, we built autoregressive integrated moving average (ARIMA) models to predict GTs for 2020 based on 2015–2019 search patterns. In addition, we estimated the proportion of searches in which COVID-19-related terms were used. To assess the potential impact of media coverage on online searches, we assessed whether weekly “asthma” GTs correlated with the number of Google News items on asthma.

**Results:**

Over the past 5 years, worldwide search volumes for asthma and COPD reached their maximum values in March 2020. Such was not observed for diabetes, hypertension and Crohn’s disease. In 38 (70%) countries, GTs on asthma were higher in March 2020 than the respective maximum predicted values. This compares to 19 countries for COPD, 23 for hypertension, 11 for Crohn’s disease, and 9 for diabetes. Queries with COVID-19-related terms represented up to 47.8% of the monthly searches on asthma, and up to 21.3% of COPD searches. In most of the assessed countries, moderate-strong correlations were observed between “asthma” GTs and the number of news items on asthma.

**Conclusions:**

During March 2020, there was a peak in searches on asthma and COPD, which was probably mostly driven by media coverage, as suggested by their simultaneity in several countries with different epidemiological situations.

## Introduction

Google Trends (GTs), a web-based surveillance tool, can provide insights into the real-life epidemiology of diseases and outbreaks. This tool provides information—on a relative scale—on how often a certain keyword or query is searched, allowing to compare different regions, time periods, or keywords. However, as GTs assess individuals’ health information-seeking behaviour, data do not often reflect the true epidemiological situation of the searched conditions [1, 2]. In fact, there are cases that describe media coverage being associated with anomalously high online interest on many health topics, such as coronary heart disease [3], pollen counts [4, 5] or COVID-19 [6, 7].

In the context of COVID-19, several GT-based studies have been conducted with the aim of assessing whether online search data correlated with the number of COVID-19 cases and deaths. Variable results have been observed [8, 9]. In addition, GTs have been used to assess variations in online searches for health topics, with particular focus on mental health and behaviour-related searches [10–14]. In fact, different studies consistently found a decrease in searches for suicide- and depression/anxiety-related terms in the initial phase of the COVID-19 pandemic [11, 12, 14]. Search patterns on respiratory diseases, however, have been less often assessed. While a preliminary study visually identified anomalous online search interest for asthma occurring simultaneously in several countries of the Northern and Southern Hemispheres (Bousquet et al., unpublished data), it is unclear as to what has been driving such unparalleled search interest, and whether similar search patterns also occur with other respiratory and non-respiratory chronic diseases. Understanding whether the perception of symptoms of chronic respiratory diseases may be masquerading those of COVID-19 [15], or whether searches are being driven mostly by users’ curiosity/concerns, may have potentially relevant implications. Such implications concern, among others: (i) the usefulness of GTs in the epidemiological monitoring of chronic diseases, (ii) the way the occurrence of COVID-19 in patients with chronic respiratory diseases is being discussed in the media, or is being communicated to patients, and (iii) the pertinence of Google providing health screening questionnaires following searches on certain expressions [16].

Therefore, in this infodemiological study, we aimed to quantify whether there was an increased search activity on two chronic respiratory diseases—asthma and chronic obstructive pulmonary disease (COPD)—in the context of the COVID-19 pandemic. In addition, we aimed to assess whether such eventual abnormal search activity (i) could also be observed in other chronic diseases, and (ii) was associated with COVID-19-related searches.

## Methods

We assessed online searches for two respiratory diseases (asthma and COPD) and three non-respiratory chronic diseases over the past 5 years up until May 31, 2020. This period includes the first months of the COVID-19 pandemic. Online searches were assessed using GTs (https://trends.google.com/; Google, LLC, Mountain View, CA, USA) for 54 countries identified by GTs as “major countries”, including 23 in Europe, 19 in Asia and the Pacific, ten in the Americas, and two in Africa. We adopted a time series approach and assessed in detail how the COVID-19 pandemic impacted search patterns on these diseases.

### Disease and keyword selection

We focused on asthma and COPD and included three non-respiratory chronic diseases (diabetes, hypertension, and Crohn’s disease) for comparison. The three non-respiratory chronic diseases were selected on the grounds that (i) diabetes and hypertension are common comorbid conditions that have been associated with a worse COVID-19 prognosis and (ii) Crohn’s disease—like asthma—is relatively frequent in young people (who are the most active Internet users), and can manifest as diarrhoea (which may also occur in COVID-19). We did not assess any other chronic disease, as GTs limit the number of simultaneously compared queries to five. In particular, we did not assess rhinitis as it does not appear to be associated with COVID-19 searches (Bousquet, submitted).

In addition, GTs for chronic respiratory diseases were plotted along GTs for acute pneumonia. Searches for acute pneumonia were used as a proxy for searches for coronavirus/COVID-19 (as the search volume for the latter is so large that comparisons with chronic diseases are impossible), since searches on these two concepts reached their maximum values at the same time throughout 2020 (Bousquet, unpublished).

On account of the selected diseases, we retrieved GTs data on the following keywords (as “topics”): “asthma”, “chronic obstructive pulmonary disease”, “diabetes”, “hypertension”, and “Crohn’s disease”. For pneumonia, the keyword “acute pneumonia” (as “topic”) was used (of note, in GTs, “topics” are groups of search terms that share the same concept [17]; “asthma”, “chronic pulmonary obstructive disease”, “Crohn’s disease” and “acute pneumonia” are classified by Google as being “disease topics”; “diabetes” as a “disorder topic”; and “hypertension” as a “medical condition topic”). Along with GTs on these keywords, we retrieved GTs data on searches involving each chronic disease and COVID-19-related terms (this allowed us to quantify how much the 2020 GTs peaks on chronic diseases were driven by COVID-19-related searches). For each country, we built a query in its native language(s), consisting of terms specific to each chronic disease along with COVID-19-related terms (Additional file 1: Table S1). Whenever available, we used top-related or rising query expressions (starting on the most popular and until the character limit was reached). In the absence of relevant top-related or rising queries, we combined the most popular terms to search for each chronic disease along with the most popular terms to search for COVID-19/coronavirus.

### Data analysis

Google Trends values represent the Google search interest over time for a given topic as a proportion of all searches on all topics on Google at that time and location. Values are indexed to 100, where 100 is the maximum search interest for the time and location selected. The values are re-indexed according to the selected time period.

We started by analysing the worldwide search interest patterns of these five chronic diseases over the past 5 years (up to May 31, 2020), to visually assess the presence of spikes during the COVID-19 pandemic. For this assessment, GTs on chronic diseases were plotted along GTs for “acute pneumonia”. As a particular case, we compared the volume of searches subsequent to the thunderstorm-asthma of Australia (November 2016) with COVID-19-associated searches in asthma, as the former was the largest “asthma” spike that had previously been retrieved worldwide [18].

Subsequently, we studied the search patterns of the five aforementioned chronic diseases during 2020 (January–May) in the 54 countries identified by GTs as “major countries”. Our aim was to assess whether the search interest values on chronic diseases in each of these countries exceeded those that would be expected based on patterns from the previous years. For this assessment, we built seasonal autoregressive integrated moving average (ARIMA) models to predict GTs for 2020 based on the GT patterns from 2015–2019. Seasonal ARIMA models are defined by the parameters (*p*, *d*, *q*)(*P*, *D*, *Q*)_*s*_, with *p* corresponding to the order of autoregression, *d* to the degree of difference, *q* to the order of the moving average part, *P* to the seasonal order of autoregression, *D* to the seasonal integration, *Q* to the seasonal moving average, and *s* to the length of the seasonal period [19] (for an example of the use of seasonal ARIMA models for health forecasting, as well as for a discussion on their methodological strengths and limitations, please consult the study of Song et al. [19]). In this study, we applied seasonal ARIMA(3,0,2)(0,1,1)_52_ models, using weekly GT data (thus explaining the length of the seasonal period—*s*—being 52). For each model, we retrieved the maximum values—for the whole year of 2020, and for the month of March—of the upper bound of 95% confidence intervals of predicted GTs (“maximum predicted values”). Such maximum values were compared with the maximum observed GTs for the year of 2020 (January–May) and for the month of March. A search peak was formally defined as any situation in which, for a given search term, the observed GT exceeded the respective maximum predicted value.

Subsequently, for the year of 2020, we considered that GTs on each of the five selected chronic diseases (i.e., total volume of searches in a given period of time) could be divided into two components: (i) searches without any COVID-19-related term, and (ii) online searches on each chronic disease along with COVID-19-related terms (i.e., “COVID-19 related-searches” estimated for each country, using the queries listed in Additional file 1: Table S1). For each month, we calculated the average proportion that the latter represented among GTs on each chronic disease. In addition, for each month, we subtracted the average GTs on each chronic disease + COVID-19-related terms from the average total GTs on each chronic disease. The difference was compared with the respective predicted value as estimated by previously described seasonal ARIMA models. This allowed us to assess whether there might be an excess of searches on asthma beyond that explained by queries including COVID-19-related terms.

Finally, to preliminarily assess the impact of media coverage on online searches, we estimated the correlations between GTs and Google News (https://news.google.com/; Google, LLC, Mountain View, CA, USA) items on asthma in 19 different countries. For each country, we retrieved the weekly number of Google News search results (i.e., searches in news items, which differ from the Google News aggregator service present in several countries) when searching the query “asthma” in the respective language and applying the respective country and language restriction filters. Unrelated results (namely those which had only been retrieved because the respective websites advertised news for asthma) were not counted. Correlations were estimated by computing Pearson correlation coefficients.

Data analysis was performed using software R version 4.0.0 (R Foundation for Statistical Computing, Vienna, Austria).

## Results

### Five-year searches for chronic diseases

**Fig. 1:**
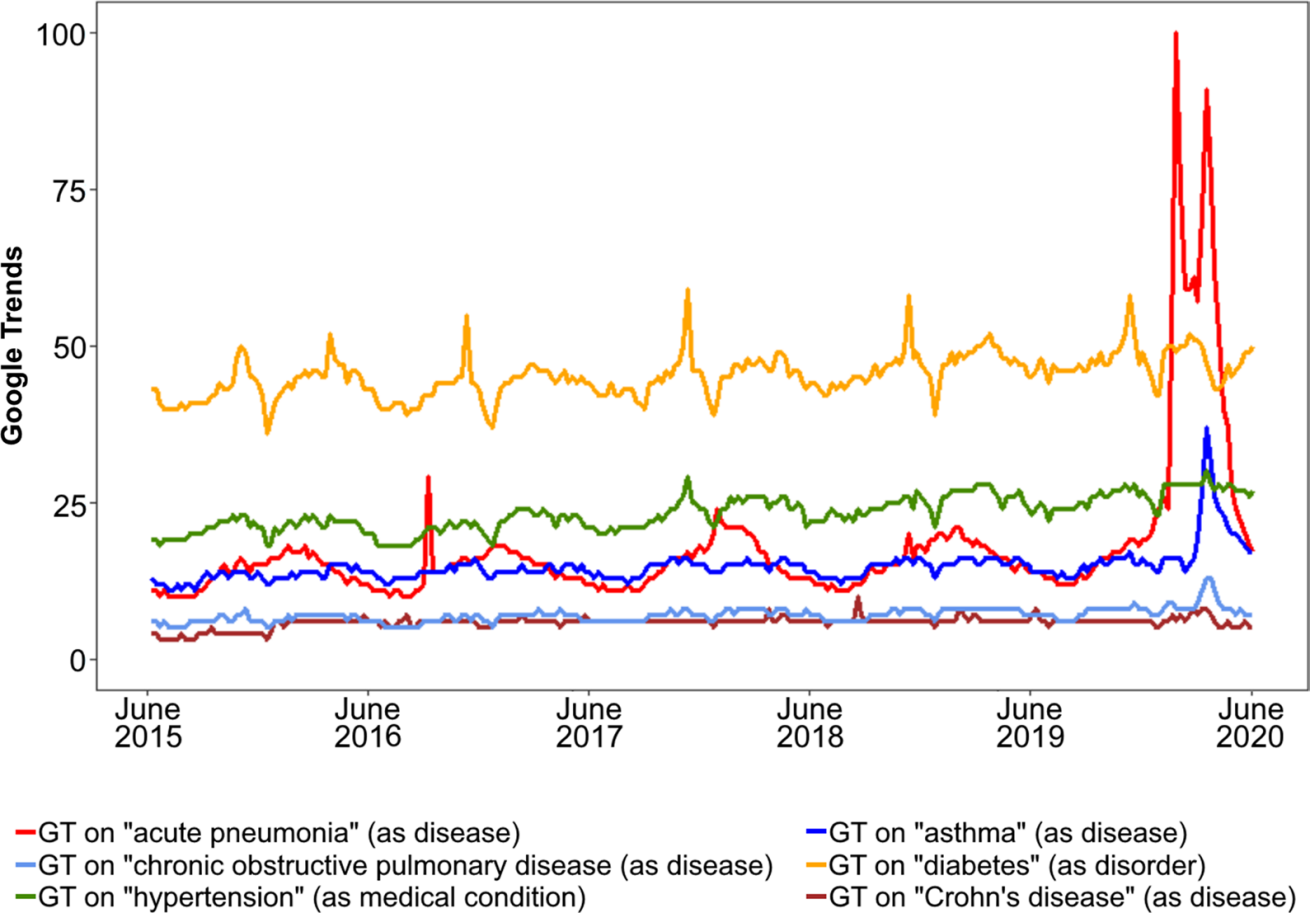
Worldwide 5-year Google Trends for respiratory and non-respiratory chronic diseases

When visually analysing 5-year data from all countries combined, we observed that asthma and (on a lesser scale) COPD searches reached their maximum values in March 2020, simultaneously with a search spike on acute pneumonia (Fig. 1). In Australia, the maximum volume of asthma searches in March 2020 was 23% lower than that observed in the week of November 20–26, 2016 (associated with the thunderstorm-induced asthma). On the other hand, there were only two countries where the 2020 GTs for COPD reached higher values than those for asthma: in Hungary, COPD maximum values occurred 2 weeks after those for asthma, whereas in Turkey, they occurred simultaneously (Additional file 1: Fig. S1).

For non-respiratory chronic diseases, no worldwide search spikes were visually identified in 2020 (Fig. 1). By contrast, we identified annual spikes for diabetes associated with the World Diabetes Day. Visually assessing 2020 data for each specific country (Additional file 1: Figs. S1, S2), large diabetes spikes were found for three specific countries, namely Italy (starting during the onset of the COVID-19 pandemic and having a 2-month duration), Romania (week of March 29) and Sweden (week of April 12). In these three cases, maximum 2020 GTs were greater than GTs in the “World Diabetes Day” weeks.

### Quantification of search peaks for chronic diseases in 2020

In March 2020, search peaks for asthma GTs were identified in 38 out of the 54 studied countries (70.4%) (Tables 1, 2). Such peaks were observed in the assessed countries of Europe (apart from Romania, Russia and Ukraine), in the Americas (apart from Mexico), in Australia and New Zealand, but only in one third of Asian countries. Search peaks for COPD were temporally consistent with those of asthma, but were only observed for 19 countries, mostly those located in Central Europe, North America, and the Pacific. However, COPD search peaks were overall smaller than those observed for asthma.

**Table 1:** Observed and predicted maximum values for Google Trends (GT) on five chronic diseases (January–May 2020)

	Asthma		COPD		Diabetes mellitus		Hypertension		Crohn’s disease	
	Maximum GT observed value (2020)	Maximum GT predicted value (2020)	Maximum GT observed value (2020)	Maximum GT predicted value (2020)	Maximum GT observed value (2020)	Maximum GT predicted value (2020)	Maximum GT observed value (2020)	Maximum GT predicted value (2020)	Maximum GT observed value (2020)	Maximum GT predicted value (2020)
Europe										
Austria	*67*	*33*	*42*	*38*	73	94	45	49	21	28
Belgium	*67*	*32*	*33*	*24*	83	91	41	47	28	32
Bulgaria	*30*	*28*	17	18	93	100	78	78	11	19
Czech Republic	*37*	*35*	*30*	*18*	87	92	32	35	30	43
Denmark	*87*	*30*	26	31	84	93	13	14	*18*	*16*
Finland	*76*	*38*	15	17	75	86	*31*	*28*	18	20
France	*100*	*27*	*24*	*13*	*68*	*61*	*51*	*44*	28	31
Germany	*87*	*30*	*53*	*39*	79	89	41	44	18	24
Greece	*52*	*20*	*27*	*15*	86	100	34	37	17	34
Hungary	*34*	*20*	*100*	*14*	64	68	*50*	*47*	16	20
Ireland	*100*	*36*	*45*	*31*	*84*	*78*	*49*	*43*	*29*	*28*
Italy	*41*	*29*	11	12	*100*	*32*	*74*	*68*	32	33
Netherlands	*64*	*22*	*27*	*23*	64	82	34	37	14	18
Norway	*100*	*35*	17	27	58	70	*49*	*32*	20	25
Poland	*28*	*18*	*11*	*9*	70	79	25	33	*20*	*12*
Portugal	*50*	*25*	9	15	59	75	28	31	16	24
Romania	19	22	7	10	*100*	*97*	*42*	*31*	6	11
Russia	26	32	9	10	68	97	34	41	7	10
Spain	*62*	*21*	15	23	57	62	66	67	17	59
Sweden	*41*	*15*	14	15	*100*	*44*	*37*	*20*	7	8
Switzerland	*100*	*26*	*28*	*22*	56	59	*62*	*35*	14	21
Ukraine	24	25	*10*	*8*	91	100	39	47	11	11
United Kingdom	*100*	*13*	*35*	*13*	*58*	*43*	*30*	*18*	12	14
Africa										
Egypt	*16*	*8*	3	4	*87*	*86*	13	15	*69*	*4*
South Africa	*32*	*22*	10	11	70	72	*75*	*55*	9	9
North America										
Canada	*69*	*24*	*31*	*24*	96	100	47	55	18	24
USA	*60*	*28*	*28*	*27*	99	100	61	66	16	22
Latin America										
Argentina	*21*	*10*	7	9	30	37	30	32	3	100
Brazil	*52*	*29*	*11*	*9*	87	96	*72*	*65*	8	29
Chile	*49*	*28*	12	19	69	87	73	74	8	12
Colombia	*26*	*25*	*35*	*20*	52	63	*100*	*81*	4	25
Ecuador	23	23	12	12	56	71	*75*	*58*	*9*	*8*
Mexico	19	21	12	12	76	87	*69*	*67*	4	5
Peru	*42*	*33*	7	9	76	89	*74*	*60*	5	6
Venezuela	31	32	*13*	*12*	54	63	71	71	5	10
Asia										
Hong Kong	25	33	14	16	87	100	47	63	8	14
India	*24*	*20*	10	11	78	79	*37*	*34*	3	6
Indonesia	53	54	7	10	95	100	93	100	2	2
Iran	78	82	1	2	20	72	16	19	*45*	*4*
Israel	*27*	*22*	10	11	85	98	23	29	23	25
Japan	*70*	*48*	*12*	*9*	90	94	29	34	6	11
Malaysia	36	45	11	13	90	100	71	80	4	5
Pakistan	36	42	17	21	100	100	69	77	*8*	*7*
Philippines	*87*	*61*	20	23	90	100	*100*	*91*	6	9
Saudi Arabia	13	16	3	4	*86*	*84*	9	12	*46*	*5*
Singapore	20	22	7	10	61	81	37	86	4	7
South Korea	17	23	12	13	80	93	29	45	*32*	*13*
Taiwan	27	29	9	13	95	100	59	68	4	5
Thailand	18	21	10	12	96	100	58	62	3	3
Turkey	*65*	*35*	*100*	*26*	65	79	*46*	*31*	7	10
UAE	*22*	*21*	6	9	64	82	30	34	*12*	*9*
Vietnam	18	20	6	9	*100*	*89*	34	36	2	2
Pacific										
Australia	*77*	*53*	*28*	*25*	88	94	46	47	17	24
New Zealand	*91*	*42*	*39*	*27*	76	100	34	52	*35*	*28*

Maximum predicted values correspond to the maximum values of the upper bound of the 95% confidence intervals for predicted asthma GT. Numbers in italic indicate cases in which maximum GT observed values were higher than maximum predicted values

*COPD* Chronic Obstructive Pulmonary Disease, *UAE* United Arab Emirates, *USA* United States of America

**Table 2:** Observed and predicted maximum values for Google Trends (GT) on five chronic diseases (March 2020)

	Asthma		COPD		Diabetes mellitus		Hypertension		Crohn’s disease	
	Maximum GT observed value (March 2020)	Maximum GT predicted value (March 2020)	Maximum GT observed value (March 2020)	Maximum GT predicted value (March 2020)	Maximum GT observed value (March 2020)	Maximum GT predicted value (March 2020)	Maximum GT observed value (March 2020)	Maximum GT predicted value (March 2020)	Maximum GT observed value (March 2020)	Maximum GT predicted value (March 2020)
Europe										
Austria	*67*	*29*	*42*	*37*	71	94	45	45	21	26
Belgium	*67*	*30*	*21*	*20*	69	88	38	42	25	27
Bulgaria	*27*	*26*	*17*	*14*	86	97	*78*	*75*	6	17
Czech Republic	*37*	*35*	14	17	63	92	32	34	18	40
Denmark	*87*	*27*	26	31	82	92	13	14	14	14
Finland	*76*	*34*	10	15	67	77	*31*	*27*	13	17
France	*100*	*25*	*24*	*12*	*68*	*60*	*51*	*44*	22	31
Germany	*87*	*30*	*53*	*34*	68	88	41	44	18	20
Greece	*52*	*20*	*27*	*15*	73	93	31	33	13	32
Hungary	*34*	*18*	*100*	*12*	37	65	39	47	10	20
Ireland	*100*	*29*	*45*	*28*	*84*	*78*	*49*	*40*	*29*	*28*
Italy	*41*	*29*	11	12	*78*	*32*	*74*	*67*	13	32
Netherlands	*64*	*21*	*27*	*21*	60	74	29	37	14	15
Norway	*100*	*28*	11	23	55	70	*49*	*32*	14	23
Poland	*28*	*18*	*11*	*8*	44	78	24	33	10	12
Portugal	*50*	*25*	9	15	52	70	18	26	15	22
Romania	18	18	7	9	*100*	*94*	*42*	*31*	4	11
Russia	26	29	9	10	61	97	33	41	6	9
Spain	*62*	*19*	15	23	56	61	*65*	*64*	13	39
Sweden	*41*	*14*	12	15	41	44	*32*	*20*	7	8
Switzerland	*100*	*26*	*28*	*22*	56	59	*62*	*34*	14	21
Ukraine	24	25	*10*	*8*	75	100	37	47	7	10
United Kingdom	*100*	*13*	*35*	*12*	*58*	*42*	*30*	*18*	12	14
Africa										
Egypt	8	8	3	4	55	86	10	14	*69*	*4*
South Africa	*32*	*20*	9	11	60	70	40	44	7	8
North America										
Canada	*69*	*23*	*31*	*24*	95	100	46	55	18	22
USA	*60*	*28*	*28*	*24*	99	100	59	64	16	22
Latin America										
Argentina	*21*	*8*	6	8	30	35	*30*	*27*	3	19
Brazil	*52*	*25*	*11*	*8*	87	96	*72*	*59*	8	25
Chile	*49*	*26*	11	17	64	87	64	69	8	10
Colombia	*26*	*22*	*35*	*19*	46	62	*100*	*74*	3	22
Ecuador	*23*	*18*	7	10	46	63	*75*	*49*	*9*	*8*
Mexico	19	19	12	12	76	87	*69*	*67*	3	5
Peru	*39*	*26*	5	9	64	87	*57*	*51*	4	6
Venezuela	*31*	*28*	5	12	48	63	71	71	3	10
Asia										
Hong Kong	23	33	14	15	69	100	43	63	5	14
India	*22*	*19*	10	10	61	79	31	33	3	4
Indonesia	53	53	7	10	87	100	83	100	1	2
Iran	68	82	1	2	16	34	16	16	*6*	*4*
Israel	*27*	*20*	9	10	52	98	18	25	19	20
Japan	*51*	*43*	*12*	*8*	68	88	26	30	4	9
Malaysia	35	40	11	13	90	100	59	79	2	5
Pakistan	30	37	12	18	66	100	55	69	7	7
Philippines	*87*	*59*	20	20	78	100	81	91	5	7
Saudi Arabia	13	16	2	3	59	83	8	12	*46*	*5*
Singapore	19	20	7	9	47	78	37	81	3	7
South Korea	17	20	10	12	63	92	27	45	7	11
Taiwan	24	29	9	12	82	100	45	68	2	5
Thailand	16	20	10	12	81	100	40	56	3	3
Turkey	*65*	*34*	*100*	*22*	60	76	*40*	*30*	6	10
UAE	*22*	*18*	6	6	55	73	22	33	6	8
Vietnam	16	20	9	9	*95*	*88*	29	36	1	2
Pacific										
Australia	*77*	*48*	*28*	*24*	85	94	*45*	*42*	17	23
New Zealand	*91*	*36*	*39*	*25*	76	100	33	42	19	28

Maximum predicted values correspond to the maximum values of the upper bound of the 95% confidence intervals for predicted asthma GT. Numbers in italic indicate cases in which maximum GT observed values were higher than maximum predicted values

*COPD* Chronic obstructive pulmonary disease, *USA* United States of America

Peaks for non-respiratory chronic diseases were not as geographically and/or temporally consistent as those for asthma or COPD. Throughout 2020, the monthly average of GTs on hypertension exceeded the predicted values in 23 countries (42.6%), mostly those in Europe (*n* = 12) and Latin America (*n* = 6). However, most GT peaks occurred in April or May (*n* = 14), including those observed in all Latin American countries. Peaks for diabetes mellitus and Crohn’s disease were observed in fewer countries (*n* = 9 and *n* = 11, respectively), and were highly variable depending on their region, month, and magnitude (of note, Crohn’s disease peaks were frequently identified in Middle Eastern countries, a fact that might be related to typos in users’ queries, given the similitude of the Arabic and Farsi words for “Crohn” and “corona”).

### Disentangling chronic diseases and COVID-19 searches

Out of the 38 countries in which asthma search peaks were identified, 28 (73.7%) had top-related or rising-related queries involving COVID-19-related terms. On the other hand, this occurred in five out of 22 (23%) countries for COPD, 11 out of 23 (48%) for hypertension, five out of nine (56%) for diabetes, and in 0 out of 11 for Crohn’s disease.

**Fig. 2:**
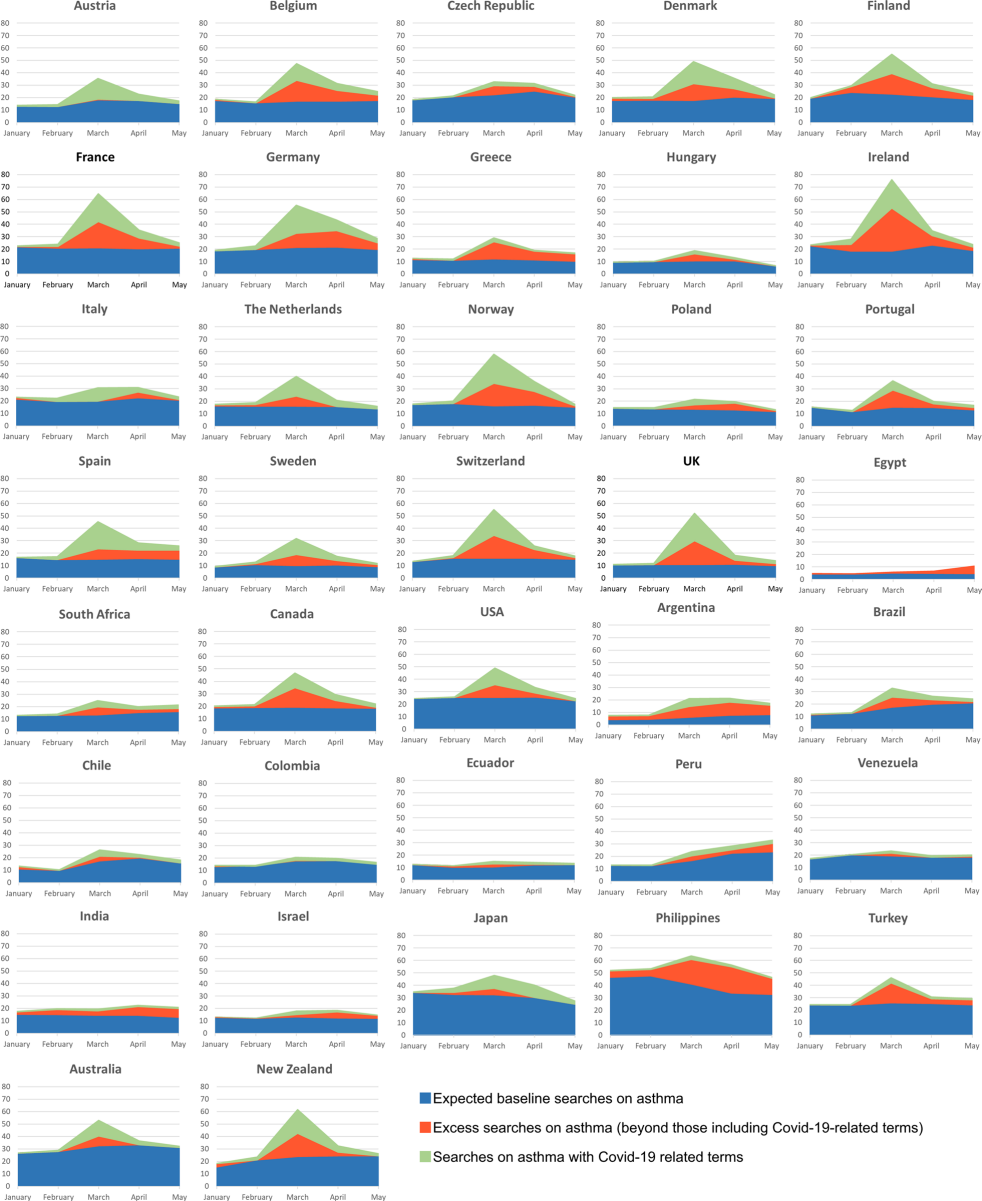
Monthly average Google Trends for “asthma” (as a disease) between January and May of 2020

In March 2020, asthma COVID-19-related searches were detected in all countries except Egypt, representing between 4.4% (for the Philippines and India) and 47.8% (for Spain) of the GTs on that disease (Table 3; Fig. 2). Overall, the percentage of COVID-19-related searches was higher in European countries, reaching over 40% in six of them. By contrast, apart from New Zealand, the percentage of COVID-19-related searches did not exceed 30% in any of the non-European countries. We also observed a variable excess of searches on asthma beyond those explained by queries including COVID-19-related terms. In March 2020, such an excess represented between 0% (for Italy) and 47.9% (for Greece) of the GTs on “asthma”.

**Table 3:** Expected and excess Google Trends on asthma and chronic obstructive pulmonary disease (COPD)

	Expected baseline searches on asthma (%)	Excess searches on asthma beyond those including Covid-19-related terms (%)	Searches on asthma with Covid-19-related terms (%)	Expected baseline searches on COPD (%)	Excess searches on COPD beyond those including Covid-19-related terms (%)	Searches on COPD with Covid-19-related terms (%)
Europe						
Austria	52.0	1.6	46.4	75.5	6.6	17.9
Belgium	36.1	35.7	28.2	65.6	21.3	13.1
Bulgaria	–^a^	–^a^	–^a^	44.9^b^	55.1^b^	0^b^
Czech Republic	68.7	22.7	8.6	30.2^c^	69.8^c^	0^c^
Denmark	36.5	27.5	36.0	–^a^	–^a^	–^a^
Finland	41.6	30.3	28.1	–^a^	–^a^	–^a^
France	32.9	32.9	34.2	41.1	41.6	17.3
Germany	38.8	20.4	40.8	53.1	28.8	18.1
Greece	41.7	47.9	10.4	49.6	44.1	6.3
Hungary	56.8	29.4	13.8	21.8	78.2	0
Ireland	24.2	45.5	30.3	57.1	29.7	13.2
Italy	65.4	0	34.6	–^a^	–^a^	–^a^
Netherlands	40.3	20.3	39.4	58.8	19.9	21.3
Norway	28.5	31.3	40.2	–^a^	–^a^	–^a^
Poland	62.5	16.2	21.3	69.6	30.4	0
Portugal	41.8	38.5	19.8	–^a^	–^a^	–^a^
Spain	33.6	18.6	47.8	–^a^	–^a^	–^a^
Sweden	31.4	28.3	40.3	–^a^	–^a^	–^a^
Switzerland	29.1	33.5	37.4	67.9	32.1	0
Ukraine	–^a^	–^a^	–^a^	64.9	35.1	0
United Kingdom	20.8	37.3	41.9	43.2	40.2	16.6
Africa						
Egypt	42.8^c^	57.2^c^	0^c^	–^a^	–^a^	–^a^
South Africa	54.0	26.6	19.4	–^a^	–^a^	–^a^
North America						
Canada	41.4	33.7	24.9	79.1	15.5	5.4
USA	51.0	20.6	28.4	89.9	2.8	7.3
Latin America						
Argentina	28.9	41.7	29.4	–^a^	–^a^	–^a^
Brazil	53.7	25.3	21.0	78.5	16.9	4.6
Chile	66.8	14.9	18.3	–^a^	–^a^	–^a^
Colombia	83.5	5.8	10.7	51.2^b^	40.3^b^	8.5^b^
Ecuador	71.5	16.5	12.0	–^a^	–^a^	–^a^
Peru	71.8^c^	20.3^c^	7.9^c^	–^a^	–^a^	–^a^
Venezuela	83.3	9.9	6.8	89.1	10.9	0
Asia						
India	64.5 ^b^	31.1^b^	4.4^b^	–^a^	–^a^	–^a^
Israel	65.7^b^	34.3^b^	3.2^b^	–^a^	–^a^	–^a^
Japan	67.7	11.0	21.3	63.4^b^	24.1^b^	12.5^b^
Philippines	64.6	31.0	4.4	–^a^	–^a^	–^a^
Turkey	56.3	37.2	6.5	38.1	58.4	3.5
Pacific						
Australia	62.0	14.9	23.1	78.8	16.1	5.1
New Zealand	38.9	30.5	30.6	52.8	47.2	0

Percentages of Google Trends on asthma and COPD corresponding to (i) expected baseline searches, (ii) excess searches beyond those including Covid–19-related terms, and (iii) searches with Covid-19-related terms. Unless otherwise indicated, search peaks were observed in March

*USA* United States of America

^a^No search peak observed (of note, no search peak for either asthma or COPD was observed for Hong Kong, Indonesia, Iran, Malaysia, Mexico, Pakistan, Romania, Russia, Saudi Arabia, Singapore, South Korea, Taiwan, Thailand, United Arab Emirates or Vietnam)

^b^Search peak occurred in April

^c^Search peak occurred in May

**Fig. 3:**
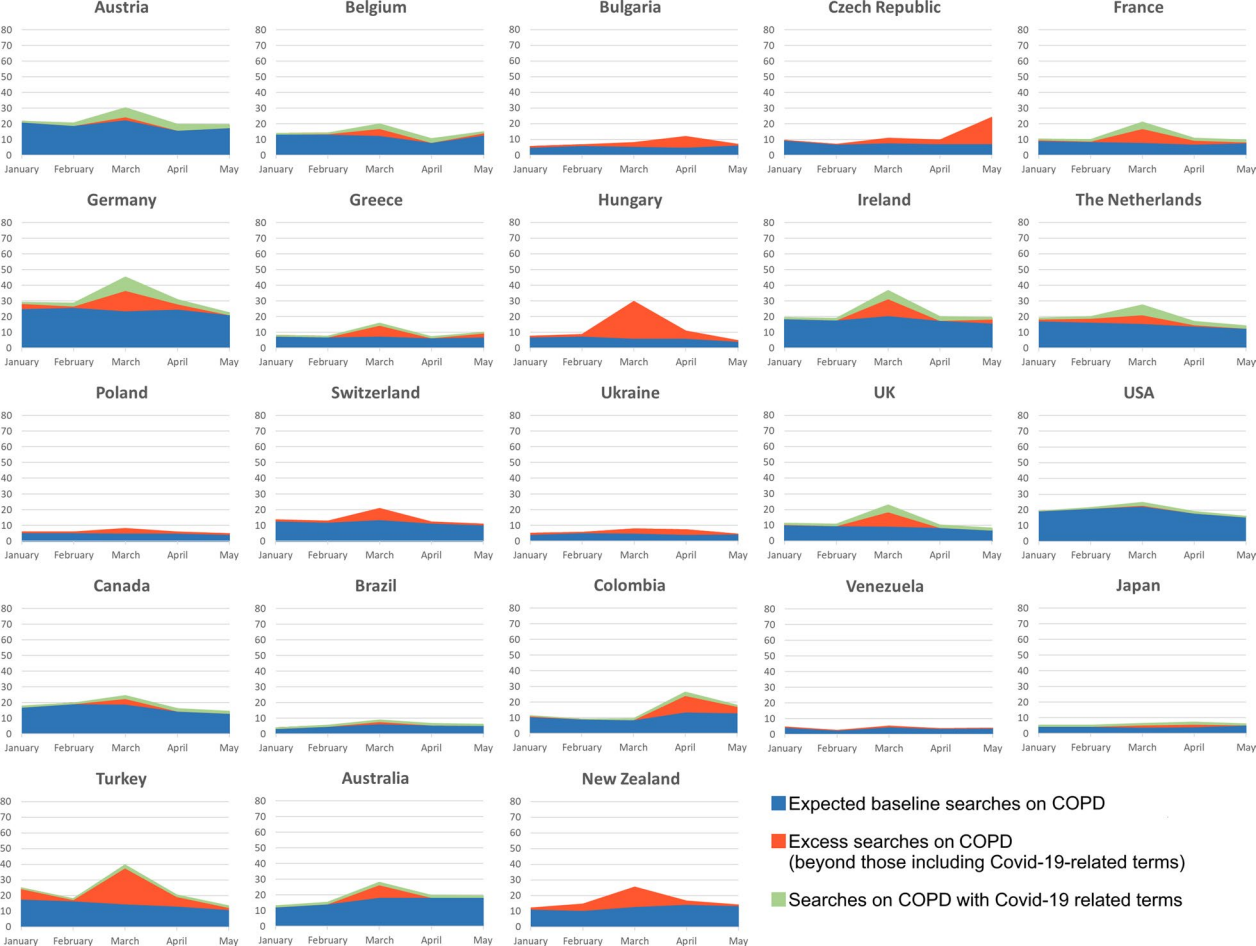
Monthly average Google Trends for “chronic obstructive pulmonary disease” (COPD) (as a disease) (2020, January–May)

The maximum percentage of COVID-19-related searches was 21.3% for COPD (the Netherlands) (Table 3; Fig. 3). For non-respiratory chronic diseases, 20.2% was reached for diabetes (United Kingdom), 20.5% for hypertension (Switzerland), and 4.7% for Crohn’s disease (Egypt) (Additional file 1: Table S2, Figs. S2–S4). The number of countries for which COVID-19-related search represented  < 1% of all GTs was eight for COPD, compared to four for diabetes, five for hypertension, and nine for Crohn’ disease.

In most countries, the number of Google News items on asthma reached their maximum value in March 2020 (Additional file 1: Fig. S5). The subsequent pattern was less consistent across countries, the number of asthma news remaining high in some and decreasing in others (often with new rises). Therefore, while GTs and Google News displayed moderate-strong correlations for the whole of 2020 (often reaching their maximum values in the same week), such correlations were stronger when specifically assessing the months of January to March (Additional file 1: Table S3). While weaker correlations were found for countries in which small or no GT asthma peaks were observed (e.g., Italy and South Korea), this was not always the rule (as suggested for the correlations observed in Colombia and the US).

## Discussion

In this study, we found that, during the COVID-19 pandemic in 2020, there was a consistent increase in web searches on “asthma” observed in several countries, particularly in March. Such an increase was variably associated with information-seeking on asthma and COVID-19 simultaneously, and resembled the thunderstorm-induced asthma-related searches in Australia (i.e., no other situation over the past years has prompted such an increase of asthma searches in all countries). Smaller and less frequent search peaks were observed for COPD, with the role of queries involving COVID-19-related terms appearing to be smaller. Such increased search activity was not consistently observed for the assessed non-respiratory chronic diseases.

Asthma primarily affects the respiratory system, and some of the main symptoms of COVID-19 are also respiratory. This fact, along with a relative lack of information (particularly when compared to diabetes or hypertension) on whether asthma can be associated with a worse prognosis of COVID-19 [20], may partly explain the particularly evident search increase observed for asthma. Another possible explanation concerns the fact that young adults, and especially parents, are particularly active Internet users [21]. In fact, asthma is relatively common among young adults and even more among children (in relation to whom, parents may wish to seek health information). Such an increase may be smaller for COPD as the latter (i) is more frequent at a more advanced age (with the elderly being less active on the Internet than the younger [1]), and (ii) is less known among the general public. In fact, regarding COPD, some of the most frequent top-related queries consisted of just asking what COPD was (data not shown).

This study suggests that GTs alone may be inadequate for prospectively assessing the epidemiology of chronic diseases, and questions Google’s strategy of displaying screening questionnaires when searching for key expressions [16]. In fact, in this study, we found that asthma search peaks occurred simultaneously in several countries in the Northern and Southern hemispheres, irrespective of the COVID-19 epidemiological situation (as suggested by the relatively small Italian search peak) or of environmental phenomena. This suggests that media coverage plays a major role in influencing GTs, as corroborated not only by the moderate-strong correlations observed with the frequency of Google News items (which should be carefully interpreted, as the amount of news does not necessarily reflect their impact), but also by the observation of search spikes related to health awareness campaigns (e.g., World Diabetes Day), or celebrity-related events. As an example, the death of the Swedish TV presenter Adam Alsing on April 15 2020—who died of COVID-19 and was known to be at risk of developing diabetes [22]—prompted the largest Swedish number of searches on diabetes of the past 5 years (observed on April 15–17). The largest Turkish GTs on COPD (occurring in the second quarter of March) also appear to be related to the death of the Turkish commander Aytaç Yalman (who suffered from COPD) on March 15 [23], as well as to the widely mediatized statements by respiratory clinicians, including members of the Turkish Thoracic Society [24, 25].

This study has important limitations that are worth discussing. Firstly, we limited our comparison to five chronic diseases, as GTs are provided on a relative scale (i.e., on a 0 to 100 scale, with 100 corresponding to the maximum volume of searches registered for the included keywords in the selected location and period of time) and do not allow the comparison of more than five queries simultaneously. However, we tried to select chronic conditions whose symptoms could masquerade those of COVID-19 or which are widely known to be associated with a worse COVID-19 prognosis. Additional limitations concern the queries used for retrieving GTs on searches involving both chronic diseases and COVID-19-related search terms, and which could have resulted in an underestimation of the percentage of searches that were COVID-19-related. In fact, due to the GT limitation of characters, we were not able to build queries using every combination of chronic disease and COVID-19-related terms. For the cases in which we were not able to include all relevant top-related and/or rising queries, we made sure that we selected the most popular ones. On the other hand, for countries in which no relevant top-related or rising queries were available, we had to build expressions ourselves, combining both chronic diseases and COVID-19-related terms. While important search variations might have been missed (particularly in countries whose native language is not fluently spoken by any of the authors of this manuscript), the impact of missing those expressions is not expected to be particularly large, as otherwise they would have been listed as top-related or rising queries. Finally, an important GT limitation concerns the geographical and demographic representativeness of Internet users. In fact, Internet use is still highly asymmetrical across different regions of the globe. Of the 54 countries identified by GTs as “major countries”, only two are located in Africa, which is home to one-seventh of the world population. In addition, in each country, the elderly (among whom diseases such as COPD, hypertension or diabetes are more frequent) are particularly underrepresented among Internet users [1], and literacy may also influence the topics of online searches.

This study also has relevant strengths. We assessed over 50 countries worldwide and took a 5-year period into account. In addition, we applied a time series approach to estimate whether the number of observed searches was higher than that predicted based on the data of previous years. Finally, we quantified the proportion of excess searches that may be related to COVID-19.

In conclusion, this study suggests that, during the COVID-19 pandemic, there was an anomalous increase in online searches on chronic respiratory diseases, which was partly accounted for by searches on COVID-19-related terms. There was also a less evident peak for COPD. Such peaks were not regularly observed for other chronic diseases. This study points to the inadequacy of GTs as an isolated tool to assess the epidemiology of chronic diseases (and, most notably, to assess it prospectively), as search patterns can be highly influenced by users’ concerns and media coverage.

## Supplementary information

**Additional file 1: Table S1.** Queries used to retrieve, for each country, Google Trends on searches involving both chronic diseases and Covid-19-related search terms. **Table S2.** Percentages of Google Trends on non-respiratory chronic diseases (diabetes, hypertension, and Crohn’s disease) corresponding to (i) expected baseline searches, (ii) excess searches beyond those including Covid-19-related terms, and (iii) searches with Covid-19-related terms. Unless otherwise indicated, search peaks were observed in March. **Table S3.** Pearson correlation coefficients between Google Trends (GT) and Google News items on asthma for the periods of January–May 2020 and January–March 2020. **Figure S1.** 2020 Google Trends for “acute pneumonia” (as a disease), “asthma” (as a disease), “chronic obstructive pulmonary disease” (COPD) (as a disease), “diabetes” (as a disorder), “hypertension” (as a medical condition). **Figure S2.** Monthly average Google Trends for “diabetes” (as a disorder) between January and May of 2020. **Figure S3.** Monthly average Google Trends for “hypertension” (as a medical condition) between January and May of 2020. **Figure S4.** Monthly average Google Trends for “Crohn’s disease” (as a disease) between January and May of 2020. **Figure S5.** Weekly Google Trends and Google News data on “asthma” in 19 countries.

## Data Availability

The datasets used and/or analysed during the current study are available from the corresponding author on reasonable request.

## Abbreviations

ARIMA: Autoregressive integrated moving average
COPD: Chronic obstructive pulmonary disease
GTs: Google Trends
