# Supplementary material for: Anomalous asthma and chronic obstructive pulmonary disease Google Trends patterns during the COVID-19 pandemic
Source: Clin Transl Allergy. 2020 Nov 2;10:47. doi: 10.1186/s13601-020-00352-9 (PMC7604916; doi:10.1186/s13601-020-00352-9)
Supplement: Supplementary file 1 — Additional file 1: Table S1. Queries used to retrieve, for each country, Google Trends on searches involving both chronic diseases and Covid-19-related search terms. Table S2. Percentages of Google Trends on non-respiratory chronic diseases (diabetes, hypertension, and Crohn’s disease) corresponding to (i) expected baseline searches, (ii) excess searches beyond those including Covid-19-related terms, and (iii) searches with Covid-19-related terms. Unless otherwise indicated, search peaks were observed in March. Table S3. Pearson correlation coefficients between Google Trends (GT) and Google News items on asthma for the periods of January–May 2020 and January–March 2020. Figure S1. 2020 Google Trends for “acute pneumonia” (as a disease), “asthma” (as a disease), “chronic obstructive pulmonary disease” (COPD) (as a disease), “diabetes” (as a disorder), “hypertension” (as a medical condition). Figure S2. Monthly average Google Trends for “diabetes” (as a disorder) between January and May of 2020. Figure S3. Monthly average Google Trends for “hypertension” (as a medical condition) between January and May of 2020. Figure S4. Monthly average Google Trends for “Crohn’s disease” (as a disease) between January and May of 2020. Figure S5. Weekly Google Trends and Google News data on “asthma” in 19 countries. [file 13601_2020_352_MOESM1_ESM.docx]

**Additional file 1**

**Table S1. Queries used to retrieve, for each country, Google Trends on searches involving both chronic diseases and Covid-19-related search terms.**

| **Country** | **Query** |
| --- | --- |
| A. Asthma |  |
| Europe |  |
| Austria | corona asthma + coronavirus asthma + covid asthma |
| Belgium | astma corona + corona en astma + astma coronavirus + astma covid + asthme coronavirus + asthme covid |
| Bulgaria | astma coronavirus + астма корона + астма COVID + астма коронавирус |
| Czech Republic | koronavirus a astma |
| Denmark | corona astma + astma og corona + astma coronavirus + astma covid |
| Finland | korona astma + koronavirus astma + korona ja astma + astma ja koronavirus |
| France | asthme coronavirus + asthme et coronavirus + asthmatique coronavirus + asthme covid 19 |
| Germany | asthma corona + coronavirus asthma + corona und asthma + corona bei asthma + asthma und coronavirus |
| Greece | ασθμα και κορονοιοσ |
| Hungary | koronavírus asztma |
| Ireland | coronavirus asthma + coronavirus and asthma + asthma and covid 19 |
| Italy | coronavirus e asma + coronavirus asmatici + asmatici e coronavirus + coronavirus asma |
| Netherlands | astma corona + corona en astma + coronavirus astma + astma en coronavirus |
| Norway | corona astma + korona astma + astma og corona + coronavirus astma + korona og astma |
| Poland | astma koronawirus + koronawirus a astma + astma oskrzelowa a koronawirus + astma i koronawirus |
| Portugal | asma corona + asma coronavírus + asma covid + asma coronavirus |
| Spain | coronavirus asma + asmaticos coronavirus + asmáticos coronavirus + asma corona + asma covid |
| Sweden | astma corona + astma och corona + coronavirus astma + covid 19 astma + corona asthma |
| Switzerland | asthma corona + asthma coronavirus + asthma und coronavirus + asthme coronavirus |
| United Kingdom | coronavirus asthma + coronavirus and asthma + asthma uk coronavirus + asthma covid + corona asthma |
| Africa |  |
| Egypt | الربو فيروس كورونا + coronavirus asthma |
| South Africa | coronavirus and asthma + asthma and covid 19 + asthma corona + asthma coronavirus + asthma covid |
| North America |  |
| Canada | asthma covid + asthma coronavirus + asthma covid 19 + asthma and coronavirus + asthma and covid 19 |
| USA | coronavirus asthma + covid asthma + asthma and coronavirus + asthma covid 19 + corona asthma |
| Latin America |  |
| Argentina | coronavirus asma + asmaticos coronavirus + asmáticos coronavirus + asma corona + asma covid |
| Brazil | corona virus asma + coronavirus asma + asmaticos coronavirus + asma covid + asma corona |
| Chile | coronavirus asma + asmaticos coronavirus + asmáticos coronavirus + asma corona + asma covid |
| Colombia | asma y coronavirus + asma coronavirus + asma covid + asma corona |
| Ecuador | asma y coronavirus + asma coronavirus + asma covid + asma corona |
| Peru | asma y coronavirus + asma coronavirus + asma covid + asma corona |
| Venezuela | asma y coronavirus + asma coronavirus + asma covid + asma corona |
| Asia |  |
| India | asthma coronavirus + asthma and coronavirus + asthma covid + asthma corona |
| Israel | אסטמה נגיף קורונה + אסתמה נגיף קורונה + asthma coronavirus + астма коронавирус |
| Japan | 喘息 コロナ + 喘息 薬 コロナ + 喘息 コロナ ウイルス + コロナ ウイルス 喘息 薬 + 咳 喘息 コロナ |
| Philippines | asthma coronavirus + hika coronavirus + asthma corona + hika corona + asthma corona virus |
| Turkey | corona astım + covid astım + covid-19 astım |
| Pacific |  |
| Australia | coronavirus asthma + coronavirus and asthma + covid 19 asthma + asthma and covid 19 + asthma corona |
| New Zealand | asthma and coronavirus + covid 19 and asthma + asthma coronavirus + asthma covid + asthma corona |
| B. Chronic obstructive pulmonary disease | |
| Europe |  |
| Austria | corona copd + coronavirus copd + covid copd |
| Belgium | copd corona + corona en copd + copd coronavirus + copd covid + bpco coronavirus + bpco covid |
| Bulgaria | хобб корона + хобб COVID + хобб коронавирус + хобб корона вирус |
| Czech Republic | koronavirus a chopn + chopn korona + chopn koronavirus + chopn covid |
| France | bpco coronavirus + bpco et coronavirus + bpco covid |
| Germany | copd corona + copd coronavirus + corona und copd + corona bei copd + coronavirus und copd |
| Greece | χαπ κορονοιοσ |
| Hungary | copd koronavirus + copd koronavírus + copd korona + copd covid |
| Ireland | coronavirus dpoc + coronavirus and dpoc + dpoc and covid 19 |
| Netherlands | copd corona + corona en copd + copd coronavírus + copd en coronavirus |
| Switzerland | copd corona + copd coronavirus + copd und coronavirus + bpco coronavirus |
| Ukraine | хобл коронавирус + хозл коронавирус + хобл коронавірус + хозл коронавірус |
| United Kingdom | copd coronavirus + coronavirus and copd + copd coronavírus uk + covid 19 copd |
| North America |  |
| Canada | copd covid + copd coronavirus + copd covid 19 + copd and coronavirus + copd and covid 19 |
| USA | copd coronavirus + coronavirus and copd + covid copd + copd covid 19 + corona copd |
| Latin America |  |
| Brazil | corona virus dpoc + coronavirus dpoc + coronavírus dpoc + dpoc covid + dpoc corona |
| Colombia | epoc coronavirus + epoc corona + epoc covid + epoc y coronavirus |
| Venezuela | epoc coronavirus + epoc corona + epoc covid + epoc y coronavirus |
| Asia |  |
| Japan | copd コロナ |
| Turkey | corona koah + covid koah + covid-19 koah |
| Pacific |  |
| Australia | coronavirus copd + coronavirus and copd + covid 19 copd + copd and covid 19 + copd corona |
| New Zealand | copd and coronavirus + covid 19 and copd + copd coronavirus + copd covid + copd corona |
| C. Diabetes | |
| Europe |  |
| France | diabete coronavirus + coronavirus et diabete + coronavirus diabète + coronavirus et diabète + covid et diabete |
| Ireland | diabetes and coronavirus + diabetes corona + diabetes covid + coronavirus diabetes |
| Italy | diabete coronavirus + diabete e coronavirus + diabete e corona virus + diabete corona |
| Romania | diabet coronavirus + diabet covid + diabet corona + diabetul coronavirus |
| Sweden | corona diabetes + diabetes coronavirus + corona och diabetes + corona diabetes typ 1 |
| United Kingdom | diabetes coronavirus + diabetes and coronavirus + diabetes covid + covid 19 diabetes |
| Africa |  |
| Egypt | السكر فيروس كورونا + coronavirus diabetes |
| Asia |  |
| Saudi Arabia | السكر فيروس كورونا + coronavirus diabetes |
| Vietnam | corona tiểu đường + virus corona tiểu đường + coronavirus tiểu đường + corona tieu duong |
| D. Hypertension | |
| Europe |  |
| Bulgaria | високо кръвно корона + високо кръвно коронавирус + хипертония корона + хипертония коронавирус |
| Finland | korkea verenpaine korona |
| France | hypertension coronavirus + coronavirus et hypertension + hypertension covid 19 + covid et hypertension |
| Hungary | magas vérnyomás koronavírus |
| Ireland | coronavirus hypertension + coronavirus blood pressure + corona hypertension |
| Italy | ipertensione coronavirus + ipertensione e coronavirus + pressione alta coronavirus |
| Norway | høyt blodtrykk corona |
| Romania | hipertensiune coronavirus + tensiunea arteriala coronavirus + hta coronavirus + hipertensiune corona + hta corona |
| Spain | hipertension coronavirus + hipertensión coronavirus + hipertension y coronavirus + hipertension y coronavirus |
| Sweden | högt blodtryck corona + högt blodtryck och corona + högt blodtryck coronavirus |
| Switzerland | bluthochdruck corona + hypertension et coronavirus + bluthochdruck coronavirus |
| United Kingdom | coronavirus high blood pressure + hypertension coronavirus + high blood pressure and coronavirus |
| Africa |  |
| South Africa | hypertension and covid 19 + coronavirus high blood pressure + hypertension coronavirus |
| Latin America |  |
| Argentina | hipertension coronavirus + coronavirus hipertensos + hipertension y coronavirus |
| Brazil | pressao alta covid + hipertenso coronavirus + hipertensão e coronavirus + hipertensão coronavirus |
| Colombia | hipertension coronavirus + hipertensión coronavirus + hta coronavirus + presion alta coronavirus |
| Ecuador | hipertension coronavirus + hipertensión coronavirus + hta coronavirus + presion alta coronavirus |
| Mexico | hipertension coronavirus + hipertensión coronavirus + hta coronavirus + presion alta coronavirus |
| Peru | hipertension coronavirus + hipertensión coronavirus + hta coronavirus + presion alta coronavirus |
| Asia |  |
| India | blood pressure coronavirus + hypertension coronavirus + bp high coronavirus |
| Philippines | high blood coronavirus + blood pressure coronavirus + hypertension coronavirus + hypertension corona |
| Turkey | tansiyon koronavirüs + tansiyon coronavirus + hipertansiyon koronavirüs + hipertansiyon coronavirus |
| Pacific |  |
| Australia | blood pressure coronavirus + hypertension coronavirus + hypertension corona + blood pressure corona |
| E. Crohn’s disease | |
| Europe |  |
| Denmark | crohn corona + crohns corona + crohn coronavirus + crohns coronavirus |
| Ireland | crohn coronavirus + crohns coronavirus + crohn corona + crohns corona + crohn covid + crohns covid |
| Poland | crohn koronawirus + crohn korona + crohn covid |
| Africa |  |
| Egypt | كرون فيروس كورونا + coronavirus crohn |
| Latin America |  |
| Ecuador | crohn coronavirus + crohns coronavirus + crohn covid + crohns covid + crohn corona + crohns corona |
| Asia |  |
| Iran | کرون و کورونا + کرون کورونا + کرون و ویروس کورونا + کورونا و کرون+ coronavirus crohn |
| Pakistan | coronavirus crohn + coronavirus crohn disease + corona virus crohn |
| Saudi Arabia | كرون فيروس كورونا + coronavirus crohn |
| South Korea | 코로나 바이러스 크론 병 |
| United Arab Emirates | كرون فيروس كورونا + coronavirus crohn + مرض كرون فيروس كورونا |
| Pacific |  |
| New Zealand | crohn coronavirus + crohns coronavirus + crohn corona + crohns corona + crohn corona virus |

Underlined search terms correspond to those that had not been presented in top-related or rising related queries of Google Trends

**Table S2. Percentages of Google Trends on non-respiratory chronic diseases (diabetes, hypertension, and Crohn’s disease) corresponding to (i) expected baseline searches, (ii) excess searches beyond those including Covid-19-related terms, and (iii) searches with Covid-19-related terms. Unless otherwise indicated, search peaks were observed in March.**

|  | **Diabetes mellitus** | | | **Hypertension** | | | **Crohn’s disease** | | |
| --- | --- | --- | --- | --- | --- | --- | --- | --- | --- |
|  | **Expected baseline searches (%)** | **Excess searches beyond those including Covid-19-related terms (%)** | **Searches with Covid-19-related terms (%)** | **Expected baseline searches (%)** | **Excess searches beyond those including Covid-19-related terms (%)** | **Searches with Covid-19-related terms (%)** | **Expected baseline searches (%)** | **Excess searches beyond those including Covid-19-related terms (%)** | **Searches with Covid-19-related terms (%)** |
| Europe |  |  |  |  |  |  |  |  |  |
| Bulgaria | - † | - † | - † | 100 ‡ | 0 ‡ | 0 ‡ | - † | - † | - † |
| Denmark | - † | - † | - † | - † | - † | - † | 83.4 | 16.6 | 0 |
| Finland | - † | - † | - † | 93.5 | 0 | 6.5 | - † | - † | - † |
| France | 88.8 § | 9.0 § | 2.2 § | 76.6 | 5.0 | 18.4 | - † | - † | - † |
| Hungary | - † | - † | - † | 88.3 ‡ | 11.7 ‡ | 0 ‡ | - † | - † | - † |
| Ireland | 84.4 | 0 | 15.6 | 68.3 | 15.9 | 15.8 | 73.4 | 26.6 | 0 |
| Italy | 29.7 § | 66.5 § | 3.9 § | 87.5 ‡ | 12.2 ‡ | 0.3 ‡ | - † | - † | - † |
| Norway | - † | - † | - † | 58.9 | 32.5 | 8.6 | - † | - † | - † |
| Poland | - † | - † | - † | - † | - † | - † | 64.3 ‡ | 35.7 ‡ | 0 ‡ |
| Romania | 99.3 § | 0 § | 0.7 § | 74.9 ¶ | 25.1 ¶ | 0 ¶ | - † | - † | - † |
| Spain | - † | - † | - † | 83.8 ¶ | 10.2 ¶ | 6.0 ¶ | - † | - † | - † |
| Sweden | 58.6 ¶ | 34.1 ¶ | 7.3 ¶ | 46.2 ¶ | 40.1 ¶ | 13.7 ¶ | - † | - † | - † |
| Switzerland | - † | - † | - † | 55.8 | 23.7 | 20.5 | - † | - † | - † |
| United Kingdom | 78.6 | 1.2 | 20.2 | 72.3 | 8.3 | 19.5 | - † | - † | - † |
| Africa |  |  |  |  |  |  |  |  |  |
| Egypt | 66.0 †† | 33.1 †† | 0.9 †† | - † | - † | - † | 4.7 | 90.6 | 4.7 |
| South Africa | - † | - † | - † | 61.2 †† | 35.2 †† | 3.6 †† | - † | - † | - † |
| Latin America |  |  |  |  |  |  |  |  |  |
| Argentina | - † | - † | - † | 52.3 †† | 44.7 †† | 3.0 †† | - † | - † | - † |
| Brazil | - † | - † | - † | 92.7 †† | 5.8 †† | 1.5 †† | - † | - † | - † |
| Colombia | - † | - † | - † | 79.8 ¶ | 15.3 ¶ | 4.9 ¶ | - † | - † | - † |
| Ecuador | - † | - † | - † | 57.1 ¶ | 42.9 ¶ | 0 ¶ | 81.3 § | 18.7 § | 0 § |
| Mexico | - † | - † | - † | 80.4 †† | 18.4 †† | 1.2 †† | - † | - † | - † |
| Peru | - † | - † | - † | 71.4 †† | 26.8 †† | 1.8 †† | - † | - † | - † |
| Asia |  |  |  |  |  |  |  |  |  |
| India | - † | - † | - † | 65.4 †† | 32.4 †† | 2.2 †† | - † | - † | - † |
| Iran | - † | - † | - † | - † | - † | - † | 11.1 § | 88.9 § | 0 § |
| Pakistan | - † | - † | - † | - † | - † | - † | 57.5 § | 42.5 § | 0 § |
| Philippines | - † | - † | - † | 72.5 ¶ | 25.1 ¶ | 2.4 ¶ | - † | - † | - † |
| Saudi Arabia | 84.0 †† | 15.5 †† | 0.5 †† | - † | - † | - † | 12.3 | 84.1 | 3.6 |
| South Korea | - † | - † | - † | - † | - † | - † | 32.1 ‡ | 67.9 ‡ | 0 ‡ |
| Turkey | - † | - † | - † | 74.4 | 21.8 | 3.8 | - † | - † | - † |
| UAE | - † | - † | - † | - † | - † | - † | 88.1 § | 11.9 § | 0 § |
| Vietnam | 89.2 | 10.8 | 0 | - † | - † | - † | - † | - † | - † |
| Pacific |  |  |  |  |  |  |  |  |  |
| Australia | - † | - † | - † | 95.0 †† | 2.6 †† | 2.4 †† | - † | - † | - † |
| New Zealand | - † | - † | - † | - † | - † | - † | 79.2 ‡ | 20.8 ‡ | 0 ‡ |

UAE: United Arab Emirates; USA: United States of America; † No search peak observed (of note, no search peak for non-respiratory chronic diseases was observed for Austria, Belgium, Canada, Chile, Czech Republic, Germany, Greece, Hong Kong, Indonesia, Israel, Japan, Malaysia, The Netherlands, Portugal, Romania, Russia, Singapore, Taiwan, Thailand, Ukraine, United States of America or Venezuela); ‡ Search peak occurred in January; § Search peak occurred in February; ¶ Search peak occurred in April; †† Search peak occurred in May.

**Table S3. Pearson correlation coefficients between Google Trends (GT) and Google News items on asthma for the periods of January-May 2020 and January-March 2020.**

|  | **January-May** | **January-March** |
| --- | --- | --- |
| Europe |  |  |
| Austria | 0.539 | 0.743 |
| Belgium | 0.680 | 0.790 |
| Czech Republic | 0.433 | 0.566 |
| France | 0.385 | 0.675 |
| Germany | 0.120 | 0.307 |
| Ireland | 0.701 | 0.860 |
| Italy | 0.135 | 0.184 |
| Netherlands | 0.455 | 0.825 |
| Spain | 0.525 | 0.613 |
| Sweden | 0.811 | 0.889 |
| Switzerland | 0.656 | 0.916 |
| United Kingdom | 0.164 | 0.537 |
| Americas |  |  |
| Brazil | 0.714 | 0.910 |
| Chile | 0.699 | 0.891 |
| Colombia | 0.577 | 0.890 |
| United States of America | -0.143 | 0.167 |
| Asia and Pacific |  |  |
| Australia | 0.771 | 0.825 |
| Philippines | 0.710 | 0.832 |
| South Korea | 0.178 | 0.190 |

**Figure S1. 2020 Google Trends for “acute pneumonia” (as a disease), “asthma” (as a disease), “chronic obstructive pulmonary disease” (COPD) (as a disease), “diabetes” (as a disorder), “hypertension” (as a medical condition).**

**
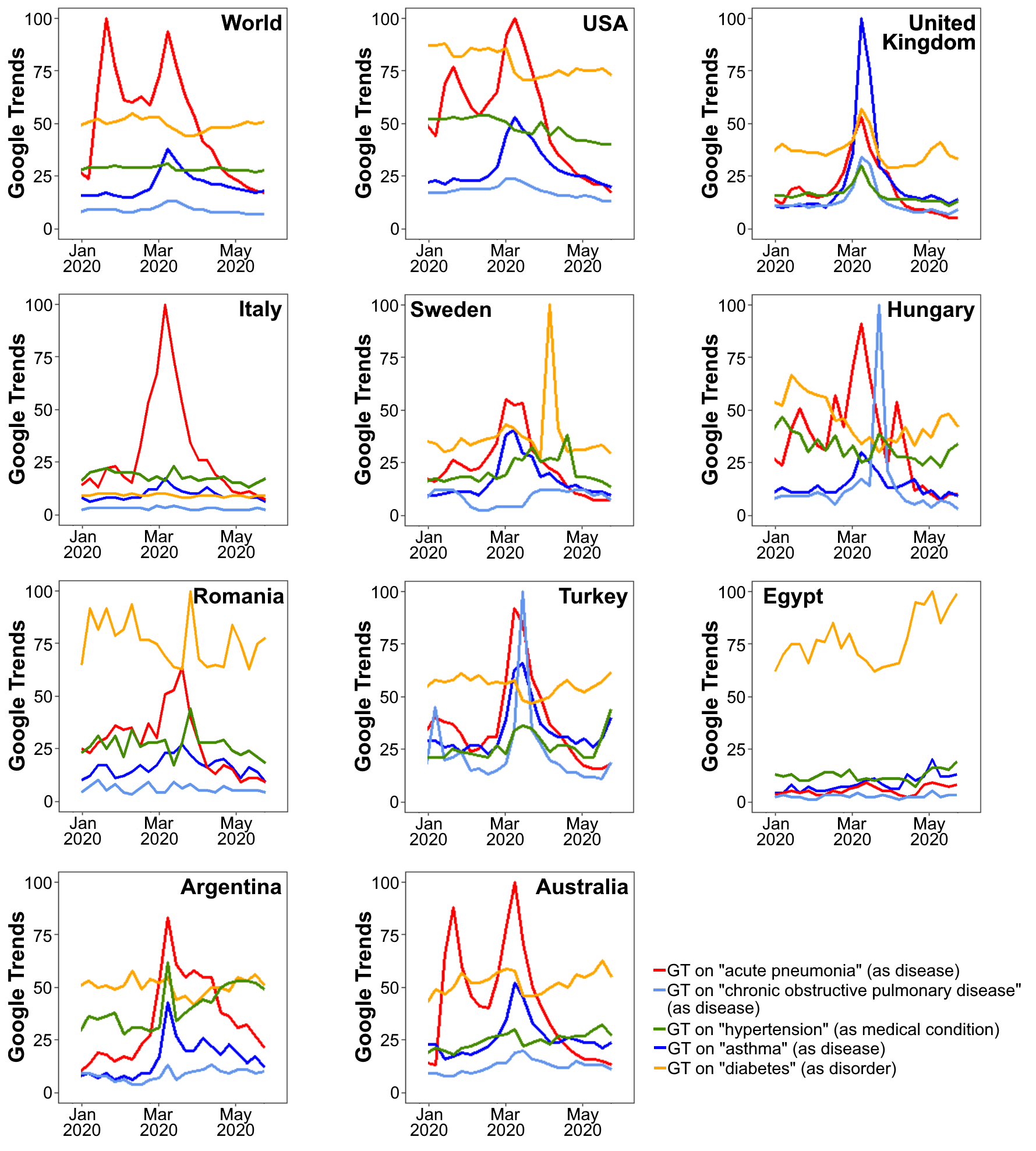
**

**Figure S2. Monthly average Google Trends for “diabetes” (as a disorder) between January and May of 2020.**

**
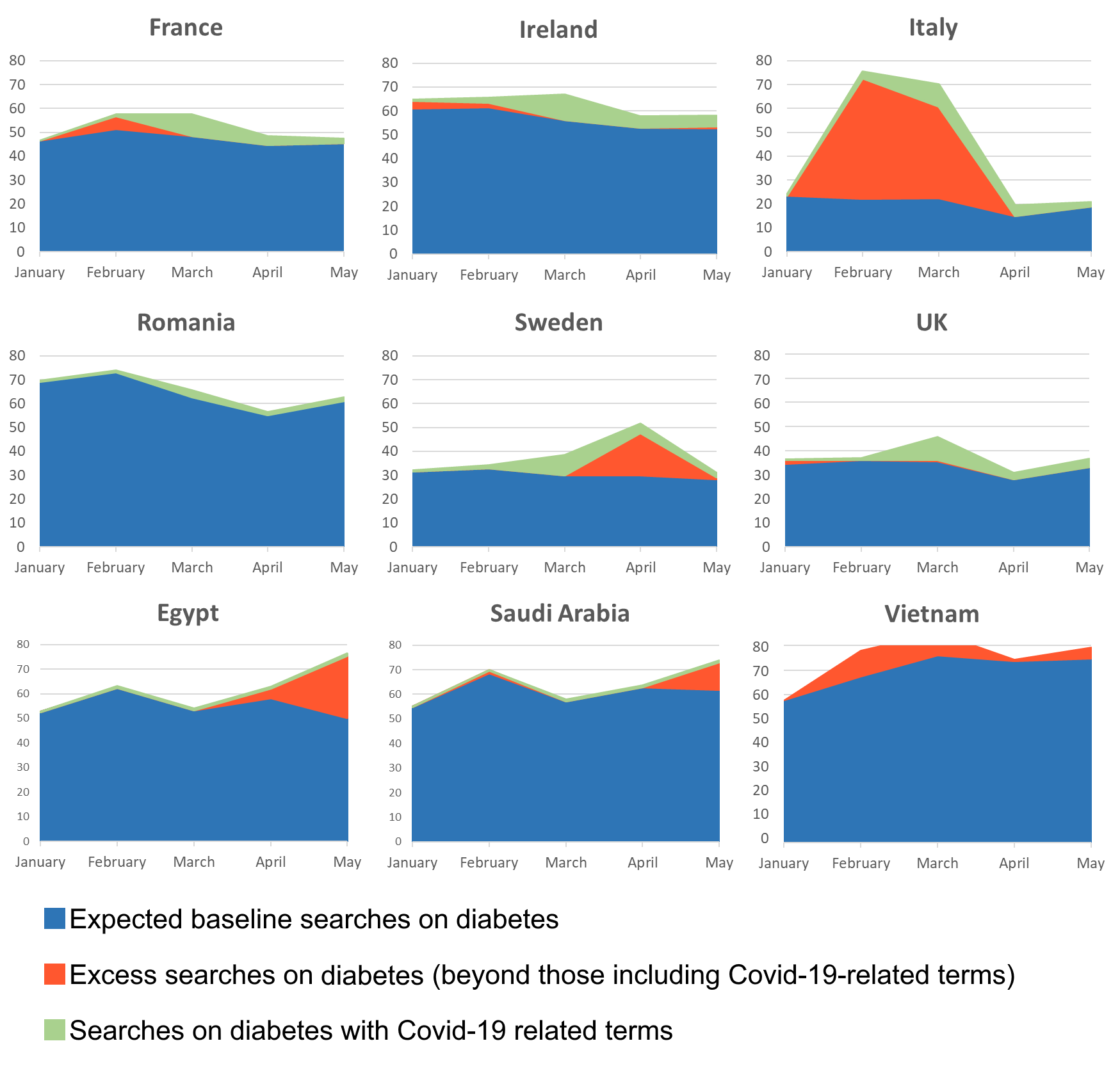
**

**Figure S3. Monthly average Google Trends for “hypertension” (as a medical condition) between January and May of 2020.**

**
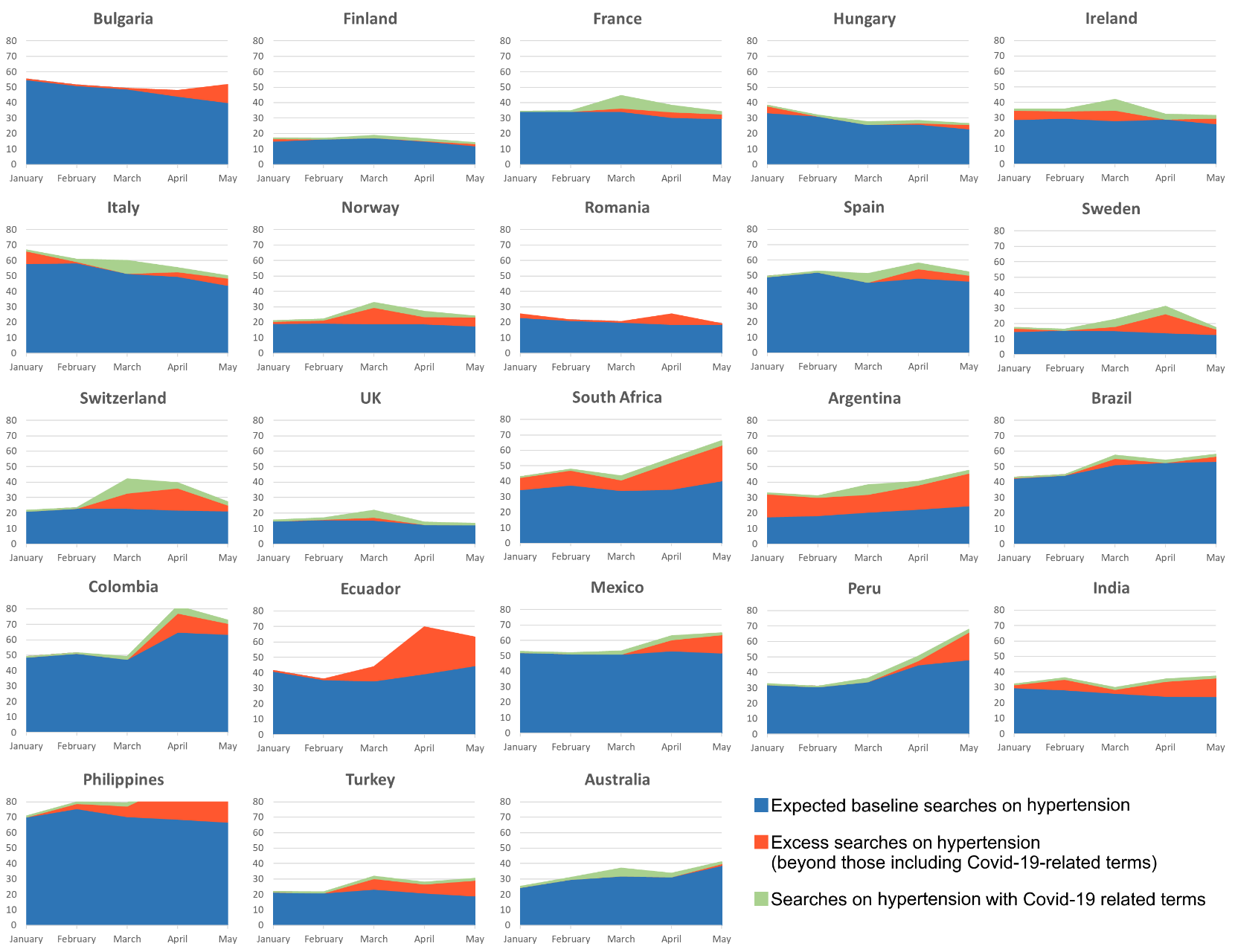
**

**Figure S4. Monthly average Google Trends for “Crohn’s disease” (as a disease) between January and May of 2020.**


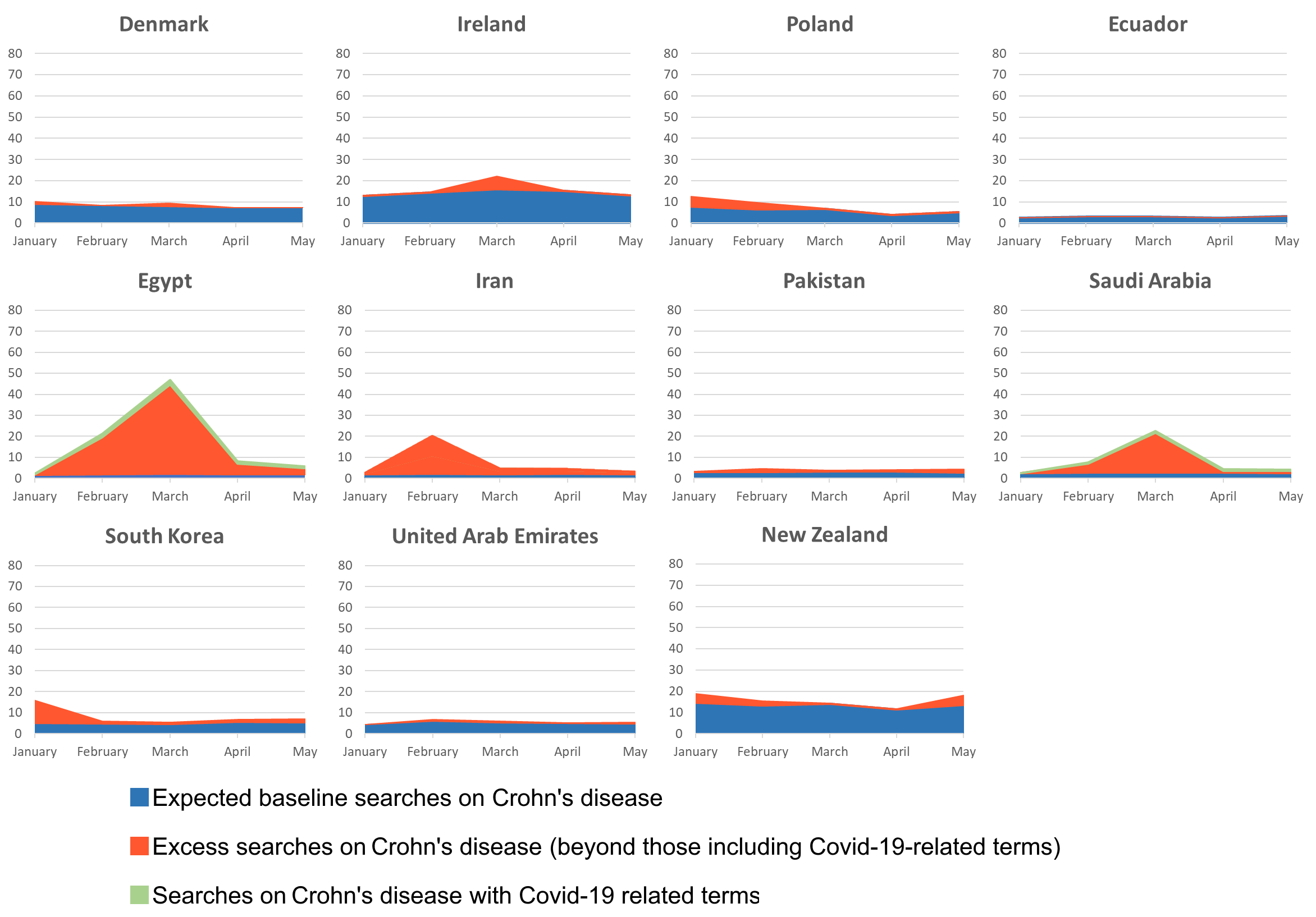


**Figure S5. Weekly Google Trends and Google News data on “asthma” in 19 countries.**

**
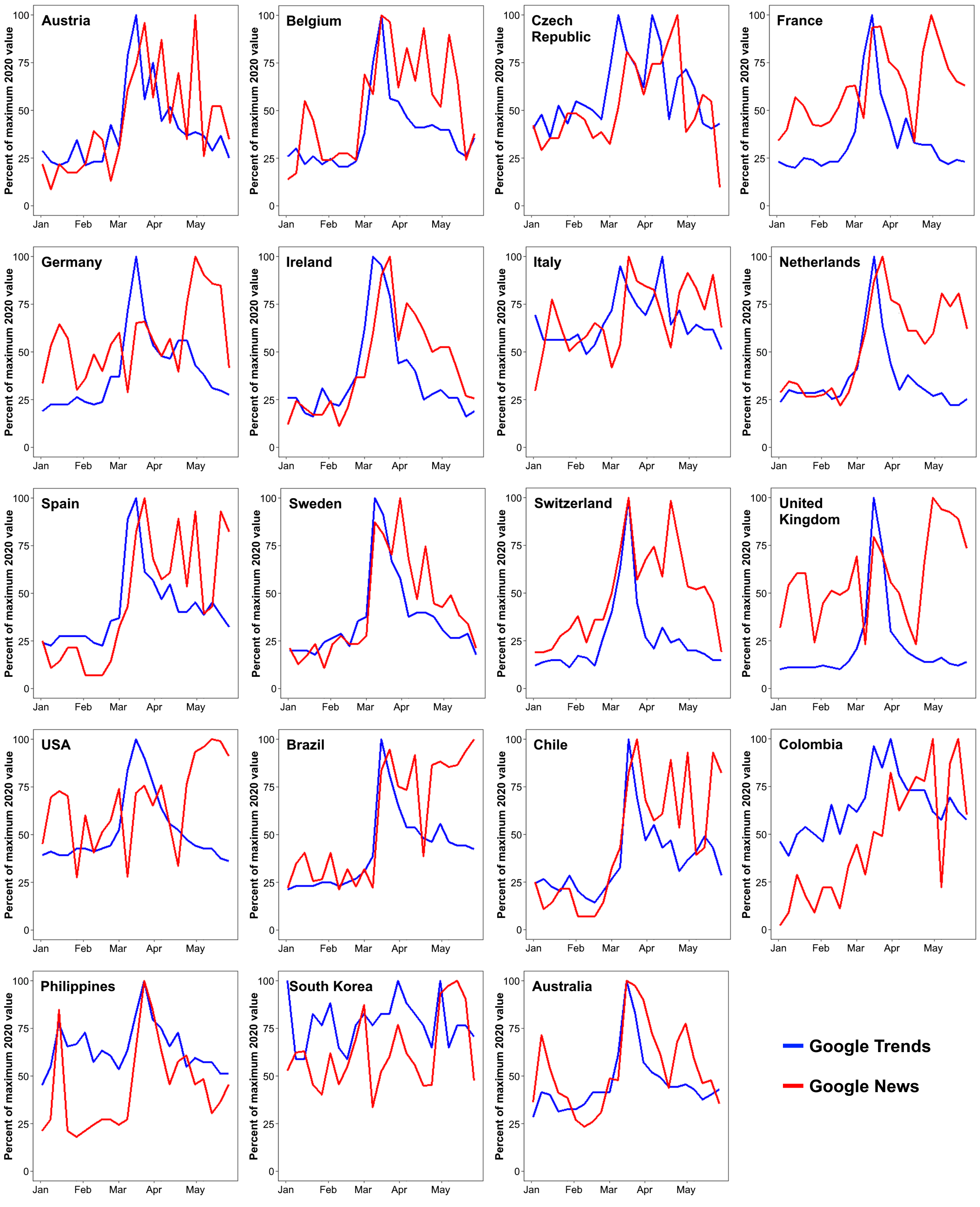
**
